# Enhancing Teacher Performance and Motivation through Effective Appraisal Policies: Evidence-Based Recommendations for Improving Educational Outcomes in Secondary Schools in Uganda.

**DOI:** 10.12688/f1000research.168834.1

**Published:** 2025-08-26

**Authors:** Asiati Mbabazi, Tukur Muhammad, Zulaiha Lawal Bagiwa, Lucy Aja

**Affiliations:** 1 Foundations, Kampala International University - Western Campus, Bushenyi, Western Region, Uganda; 2 Science Education, Kampala International University - Western Campus, Bushenyi, Western Region, Uganda

**Keywords:** Teacher Performance, Motivation, Appraisal, Policies, Evidence-Based, Recommendations, Educational Outcomes

## Abstract

Teacher performance and motivation are key drivers of quality education and student performance globally. In Uganda, secondary schools struggle with the challenges of teacher appraisal systems, and this affects students’ learning outcomes and teachers’ professional growth. An effective appraisal system is very important in supporting continuous professional development, improving accountability, and meeting international goals like Sustainable Development Goal (SDG 4). The current appraiser systems are often caused by insufficient training, limited feedback, and weak connections between performance, rewards, and barriers as a result of cultural practices that prevent honest communication. These problems destroy the appraisal system’s credibility, discourage teachers, and limit teachers’ growth. These challenges also show opportunities for the policy, such as capacity building, participatory assessments, and digital solutions, which have proven success in fostering equity, transparency, and constructive feedback. Providing training that incorporates self-assessment, peer review processes, and combining results from appraisal with incentives, thereby utilizing technology. This policy will help Uganda to create equal, more motivating, and effective systems of assessment. This policy brief promotes a culture of fairness, progressive learning, and teacher accountability, which is effective for improving teaching practices and student performance. Implementing this policy in the Ugandan education system will foster motivation among teachers, improve the standard of teaching in the country, and improve student performance. The policy will also strengthen institutional capacity, thereby improving a culture shift that moves toward open communication, and supporting Uganda’s commitment to achieving SDG 4. Also, proper monitoring is important for sustaining this policy and adapting it to changing educational needs. This policy will help in capacity building, which aims to provide a professional and motivated teaching environment that is capable of delivering a high-quality education, and such systemic change is important for achieving an inclusive, high-quality education that is aligned with global standards.

## Introduction

Globally, teaching performance and motivation are vital factors that influence the quality of education and student outcomes. Secondary schools in Uganda face continuous challenges that are associated with the appraisal systems, and this will directly impact motivation and job performance. ^1, 2^ The evidence-based policy can tackle the challenges and is important for achieving sustainable educational development that aligns with global standards. ^3^ This policy presents key results and policy recommendations to improve teacher motivation and performance through an effective appraisal system. The Performance appraisal is a systematic process for evaluating teachers’ strengths and weaknesses, and has gained increasing recognition globally as an important component of improving educational quality. ^4^ In the global context of education systems, the importance of appraisal systems, particularly in different countries that are striving for quality education and accountability, extends more than evaluation and is instrumental for promoting professional growth, accountability, which ensures that standards of education are met.

The appraisal system is rooted in its ability to improve continuous professional development (CPD). The global education system recognizes that transparent, equal, and supportive appraisal systems are important to increase teacher performance and motivation. ^4^ Such systems should be built on empirical evidence, contextually relevant, and aligned with the national development goals. This provides a structured feedback that identifies the needs of professional development, which recognizes exemplary performance, and all are essential for maintaining a high standard of teaching. ^5^ If such systems are incorporated within a wide framework of supportive supervision and professional learning, they can significantly influence the motivation and level of teachers. Research on the impact of performance appraisal systems on teachers and student outcomes is comprehensive and conclusive. According to the Organization for Economic Co-operation and Development, ^5^ Well-designed appraisal systems are correlated with improved teacher motivation, better classroom practices, and improved student learning. An appraisal systems that is ineffective weaken teachers’ intrinsic motivation, decreases job satisfaction, and boosts the rates of attrition in the teaching profession, thereby compromising education quality. ^6^ When teachers’ efforts are inadequately rewarded, their spirit of professionalism and commitment reduces, which leads to a cycle of poor performance and low student performance. This situation is globally seen in regions characterized by diverse socio-economic conditions, limited resources, and different levels of educational infrastructure. ^7^ Teachers’ experiences and perceptions of appraisal influence their motivation, which in turn affects instructional quality and student learning outcomes.

Report shows that countries with a sound communication feedback system tend to report higher levels of job and professional satisfaction among teachers. These different countries often incorporate elements such as self-assessment, peer reviews, and student feedback into their appraisal systems to create a more robust picture of teacher performance. Such practices from these different countries help to facilitate a culture of continuous improvement and fairness, which then improves the standards of teaching globally. Research further asserts that appraisal systems should not be one-sided; they must act as developmental tools that develop teachers’ growth professionally while holding them accountable for their performance. When there is an equal, focused, and constructive appraisal system as seen by teachers, it will lead to improved professional growth and significantly influence teaching practices. Studies in various countries, from Scandinavian nations to East Asian countries, show that comprehensive appraisal and feedback systems foster more reflective teaching practices, higher levels of classroom engagement, and better adaptation of teaching methods to diverse student needs. ^8^ Empirical research globally indicates that high-quality teaching is directly linked to student achievement, and the impact on student learning outcomes consistently shows that the quality of a teacher significantly influences academic performance, motivation, and long-term educational achievement. ^4^ Effective appraisal, as shown by different studies, facilitates an environment where teachers actively work on their instructional and pedagogical skills, respond to the needs of students, and utilize innovative pedagogical strategies to facilitate learning.

The Ugandan education system has improved significantly over the last few decades, this sow more children are enrolling in primary and secondary schools. Furthermore, teachers in Uganda’s secondary schools experience challenges such as not getting enough training, not getting paid enough, having too much work to perform, and not having enough opportunities to get better at their jobs. ^9^ The appraisal system is a formal technique to rate, help, and encourage teachers in their various areas of specialization, which is the most important thing to do to make teachers better. According to research, many teachers in Uganda consider performance reviews as something they have to do for the sake of the system rather than as a tool to help them progress. ^10^ Most of the time, assessments are done from the top down, and not much effort is given to how to get people to talk to each other or assist them to do better at their jobs. There is an increasing awareness in Uganda about the issues of the appraisal system, and this indicates that ineffective appraisal systems hinder the growth of professional development, motivation. Also, this calls for reforms that integrate best practices as well as strategies that are innovative in nature. Studies have shown that promoting a well-designed appraisal system that incorporates fairness, usefulness, and motivation can enhance students’ motivation and performance. ^3, 11^ This policy brief examines the issues and challenges faced in evaluating teachers in Uganda. The primary goal is to boost teacher motivation, improve the quality of instruction, and support ongoing educational growth.

Improving appraisal systems is important not only for increasing teacher motivation but also for achieving broader educational objectives such as universal access, quality learning, and equitable education. As an African country strives to meet Sustainable Development Goal 4 (SDG 4), to ensure inclusive and equitable quality education, Uganda needs to strengthen its teacher appraisal system to become a strategic imperative. ^11– 13^ This policy brief tried to outline specific approaches needed to improve the effectiveness of teacher appraisal policies, thereby contributing to better educational outcomes. Given the quest to improve the standard and quality of teaching and learning in Uganda, there is a need for teacher appraisal systems to have important tools that will improve the growth of professional development, accountability, and motivate educators. Globally, there is a recognition of the importance of appraisal systems, but many existing appraisal systems face significant challenges that decrease their effectiveness, and these challenges are effective in unlocking the full potential of appraisal systems and translating evaluation processes into meaningful improvements in teaching and student learning outcomes. One of the primary obstacles to effective teacher appraisal is insufficient training for educators and evaluators alike. Many teachers and school administrators lack a thorough understanding of appraisal procedures, criteria, and objectives, which leads to perceptions of unfairness and bias in evaluations.

Appraisal in many systems of education focuses mainly on identifying the problems and challenges without offering recommendations for the development of the appraisal, support for professional growth, or areas for improvement. Teachers are left uncertain about how to improve their performance or develop their skills, which diminishes the developmental purpose of appraisal. ^14^ Many results from appraisal do not result in meaningful rewards, promotions, or professional development opportunities, and consequently, teachers see appraisal as an evaluation that produces little importance to the progress in their career path. These views from the teachers discourage genuine effort and commitment, as teachers may not see the importance of putting more time and energy into improving their performance unless there is a clear and rewarding payoff. ^1, 15^ In addition, cultural differences, which are also a barrier, play a vital role in affecting the effectiveness of appraisal practices. In many cultures, there is respect for authority, respect for seniority, and discomfort with direct criticism alters the fairness in communication during evaluations. ^16, 17^ As a result, evaluation discussions are often superficial or overly cautious, limiting the usefulness of feedback and hindering constructive dialogue that could foster growth, while these challenges mentioned above show considerable effects, which equally present opportunities for meaningful development, and one of the most important grounds for improvement is training and capacity building. Training teachers and evaluators with the skills that are needed to conduct fair, consistent, and constructive appraisals enhances both the credibility and acceptability of the appraisal system.

Another important thing to note is to make sure that the outcomes of the appraisal align with rewards like promotions in workplaces, an increase in salaries, or special opportunities for training that can lead to professional development growth. Teachers are more likely to give their best when they believe that their hard work will be noticed and acknowledged. ^18– 21^ The moment teachers are aware of how the importance of appraisal scores can influence their professional development, the better they work on their performance. Using technology in new ways could also make the appraisal process more fun. Appraisal systems can be streamlined technologically to evaluate workflows, ensure greater transparency, facilitate real-time feedback, and maintain records efficiently. These technologies can also foster wider participation through online surveys, electronic feedback forms, and automated reminders, thereby reducing barriers and enhancing collaborations among stakeholders. Currently, teacher appraisal systems face a lot of challenges globally and not in Uganda alone, including inadequate training, limited feedback, weak salary structures, and cultural barriers; they also present significant development opportunities. This can be achieved through a capacity-building training program, participatory approaches, salary alignment, and technological innovation in education systems, which can be developed into more effective, fair, and motivating appraisal systems.

## Policy outcomes and implications

Implementation of the proposed policy in Uganda’s teacher appraisal systems promises to enhance the quality of education, and improved classroom methods, more accountability and openness, and long-term professional development are all goals of these policies. Uganda, as one of the countries in East Africa, can make its teacher appraisal system a powerful tool for professional growth and improved outcomes for students in secondary schools by organizing appraisal procedures with larger national and international objectives, like SDG 4, and guaranteeing adequate allocation of resources, capacity building, and engagement among stakeholders. Furthermore, these policies will facilitate the creation of a supportive professional learning environment that encourages reflective practice, peer collaboration, and continuous feedback. Establishing a clear link between performance assessments and incentives such as career progression, salary enhancements, and recognition will serve as additional motivation for teachers to strive for excellence. The integration of digital tools and data management systems will streamline evaluation procedures, making them more efficient and transparent, while also enabling real-time monitoring of progress and impact. Efforts to change existing cultural attitudes towards appraisal processes are equally critical. Overcoming resistance and establishing trust among community members, administrators, and educators can be achieved through the promotion of an approach that is open, participatory, and inclusive. As a result of this shift in mindset, there will be a positive feedback loop that encourages and supports educators to constantly improve their teaching methods. Last but not least, the reforms’ continued relevance, efficacy, and adaptability can be guaranteed by putting in place strong monitoring and evaluation systems. Involvement of stakeholders and modifications based on evidence will guarantee long-term reform, which will help Uganda achieve its larger objective of providing all students with access to high-quality education. With these long-term changes in place, Uganda will be able to lead the way in managing teacher performance, which will help the country’s economy and society in the long run.

## Policy recommendations for improving teacher appraisal systems

The foundation for providing all students with a high-quality education, where they can challenge their peers globally, is built on effective teacher performance and motivation, which can unlock teachers’ full potential. Context-specific, evidence-based policies should guide the development and design of current appraisal systems. Therefore, this policy brief proposes an approach that includes establishing national uniformity regulations, standardizing and professionalizing the appraisal system through a transparent and clear process, and offering training aimed at capacity-building for educators and evaluators. In order to foster a culture of ongoing professional development, it is required to employ thoughtful and interactive methods such as self- and peer-evaluation, and assign constructive rewards, and public acknowledgement with performance reviews is another proven way to boost motivation. Breaking down the cultural barriers fosters open debate grounded in trust between teachers and evaluators, whereas the integration of technology ensures that feedback is productive, transparent, and delivered in real-time. Subsequently, it is necessary to establish effective monitoring and evaluation strategies that involve stakeholders to ensure the appraisal system is fair, suitable, and reliable. Enhancing student performance and educational quality in Uganda can be accomplished through the implementation of strategic policy proposals and the transformation of teacher appraisal into an effective mechanism for professional development.

## Conclusion

Educational excellence, teacher participation, and achievement among learners are all influenced by how teacher appraisal systems work. Current systems in Uganda encounter numerous challenges that prevent them from achieving their growth goals. These challenges include inadequate training, inadequate feedback, weak financial incentives, and cultural barriers. However, opportunities for significant improvements are also presented by these challenges. Implementing evidence-based rules will significantly improve the motivation and efficiency of teachers in Uganda. Also, incorporating technology, establishing employees evaluate themselves and their peers, having performance reviews linked to promotion and rewards, and having open and participatory evaluation systems are all essential elements of effective performance management approaches. Teachers in Uganda will feel more valued, inspired, and encouraged to enhance their teaching methods on an ongoing basis if these improvements are implemented, as they will create a professional environment that is more accessible and supportive of their work. Suppose Ugandans want to raise educational standards and ensure that everyone has access to a quality education, in support of SDG 4’s associated goals. In that case, there is a need to undergo this type of change.

## Declaration

### Ethics approval and consent to participate

The study on enhancing teacher performance and motivation through effective appraisal policies, evidence-based recommendations for improving educational outcomes in secondary schools in Uganda, did not negatively impact the students, the secondary schools, or Uganda at large. The study was a thesis report and had only four objectives which was approved on 12/06/2025 by Kampala International University’s Research and Ethics Committee with approval number KIU-2024-309. Our research was conducted according to the norms and standards of the National Research Ethics Guidelines.

### Accordance

Our research was conducted according to the norms and standards of the National Research Ethics Guidelines.

## Data Availability

The researchers declared that there were no data associated with this research.
