# Supplementary material for: Enhancing Teacher Performance and Motivation through Effective Appraisal Policies: Evidence-Based Recommendations for Improving Educational Outcomes in Secondary Schools in Uganda
Source: F1000Res. 2025 Aug 26;14:826. [Version 1] doi: 10.12688/f1000research.168834.1 (PMC12824474; doi:10.12688/f1000research.168834.1)
Supplement: Peer review history

# Reviewer report: Reviewer response for version 1

**DOI:** 10.5256/f1000research.186050.r450908

**Published:** 2026-01-21

**Authors:** Tauqeer Abdullah

**Affiliations:** 1 University of Peshawar, University of Peshawar, Pakistan

Thank you for the opportunity to review this manuscript. Overall, the policy brief explores a significant area with both theoretical and practical relevance. The manuscript has strong substantive ideas, but it currently reads like an academic essay, not a policy brief. With restructuring, simplification, and sharper policy logic, it can become highly suitable for policymakers in Uganda.

**Abstract:**

 The abstract is too long for a policy brief. A policy brief abstract should read quickly and decisively for policymakers. I would be better reducing the abstract to 150-200 words. Additionally, the words like *“very important,” “effective,” “proper,”* are used frequently without analytical support. Better to replace value-laden language with policy-oriented language like “This policy aims to…”, “The proposed framework addresses…”, “Expected outcomes include…”

**Introduction:**

 The introduction is excessively long and reads like a literature review chapter. Many ideas are repeated (motivation, SDG 4, global evidence, appraisal benefits). Policymakers want context + urgency, not exhaustive theory. Authors may reduce the introduction to 3-5 short paragraphs (≈600-800 words maximum). Move detailed global evidence and cross-country examples to a short “Evidence Snapshot” section.

 The opening paragraphs are too generic and global. Uganda-specific urgency appears too late. Authors could start with Uganda first, then situate globally. Suggested policy framing logic like why teacher motivation is a policy problem in Uganda and why appraisal systems matter now.

 Authors frequently used abstract phrases like “Globally, there is a recognition…”, “It is important to note…”, “Research shows…” Introduction could be improved by replacing these phrases with policy-oriented language like “Evidence indicates…”, “Current practice in Uganda shows…”, “This creates a policy gap…” This makes the brief action-driven, not descriptive.  

**Policy outcomes and implications** **:**

 The section does not specify who benefits and how. Authors could explicitly mention implications for teachers, school leaders, ministry of education, or students. This would strengthen political and administrative relevance.

 Claiming Uganda will “lead the way” is unsupported and unnecessary. Authors could replace with realistic, policy-credible claims, e.g. “Position Uganda to strengthen accountability within its education system”.

**Conclusion:**

 The conclusion restates challenges and recommendations almost verbatim. Authors could reduce to one short paragraph that restates the policy problem, emphasizes urgency, and ends with a clear call to action. A policy conclusion should mobilize, not summarize.

Does the paper provide a comprehensive overview of the policy and the context of its implementation in a way which is accessible to a general reader?

Yes

Is the discussion on the implications clearly and accurately presented and does it cite the current literature?

Yes

Are the recommendations made clear, balanced, and justified on the basis of the presented arguments?

Yes

Reviewer Expertise:

Educational psychology, marriage and family, children and youth, gender and sexuality

I confirm that I have read this submission and believe that I have an appropriate level of expertise to confirm that it is of an acceptable scientific standard, however I have significant reservations, as outlined above.

# Reviewer report: Reviewer response for version 1

**DOI:** 10.5256/f1000research.186050.r450909

**Published:** 2026-01-20

**Authors:** Sudadi Sudadi

**Affiliations:** 1 Universitas Islam Negeri Sultan Aji Muhammad Idris Samarinda, Samarinda, East Kalimantan, Indonesia

The article presents an evidence-based policy brief on teacher appraisal systems in Ugandan secondary schools, clearly outlining the policy problem and its relevance to teacher motivation, performance, and educational quality. The discussion is accessible to a general reader and supported by largely current and relevant literature. The implications are logically derived and accurately presented, and the recommendations are clearly stated and generally justified by the preceding arguments.

 However, the paper only partially provides a comprehensive implementation context. Greater clarity is needed regarding the existing appraisal framework in Uganda and the institutional actors involved. In addition, the recommendations would benefit from a more balanced discussion that acknowledges potential implementation challenges, resource constraints, and contextual limitations. Addressing these minor points will strengthen the paper’s analytical balance, policy relevance, and overall clarity without requiring substantial revision of its core arguments.

Does the paper provide a comprehensive overview of the policy and the context of its implementation in a way which is accessible to a general reader?

Partly

Is the discussion on the implications clearly and accurately presented and does it cite the current literature?

Yes

Are the recommendations made clear, balanced, and justified on the basis of the presented arguments?

Yes

Reviewer Expertise:

education, policy education, management education

I confirm that I have read this submission and believe that I have an appropriate level of expertise to confirm that it is of an acceptable scientific standard.

# Reviewer report: Reviewer response for version 1

**DOI:** 10.5256/f1000research.186050.r442374

**Published:** 2026-01-06

**Authors:** Samra Maqbool

**Affiliations:** 1 Beijing Normal University, Beijing, Beijing, China

1. The manuscript presents a well-structured policy proposal focused on enhancing teacher performance and motivation in Uganda.
2. The connection between effective appraisal systems and improved teacher performance and student outcomes is well established.
3. The study would benefit from more empirical evidence linking specific appraisal methods to measurable improvements in teacher motivation and student performance in Uganda.
4. The discussion on cultural barriers is relevant, but more concrete examples or case studies would strengthen the argument and illustrate how these barriers can be overcome.
5. The proposal for using technology in teacher appraisals is valuable, but it needs more detail on specific tools or platforms for implementation.
6. The policy recommendations are solid, but clearer implementation steps and timelines are needed to ensure successful adoption and execution
7. A more robust evaluation framework for monitoring and assessing the impact of the proposed changes would enhance the manuscript’s practical applicability.

Does the paper provide a comprehensive overview of the policy and the context of its implementation in a way which is accessible to a general reader?

Yes

Is the discussion on the implications clearly and accurately presented and does it cite the current literature?

Yes

Are the recommendations made clear, balanced, and justified on the basis of the presented arguments?

Yes

Reviewer Expertise:

Education

I confirm that I have read this submission and believe that I have an appropriate level of expertise to confirm that it is of an acceptable scientific standard, however I have significant reservations, as outlined above.
